# The Multi-System Roles of Dp71 Dystrophin Isoforms in Duchenne Muscular Dystrophy

**DOI:** 10.3390/muscles5020043

**Published:** 2026-06-11

**Authors:** Harry Wilton-Clark, Alishba Raza, Toshifumi Yokota

**Affiliations:** 1 Department of Medical Genetics, Faculty of Medicine and Dentistry, University of Alberta, Edmonton, AB T6G 2H7, Canada; 2 Department of Biological Sciences, Faculty of Science, University of Alberta, Edmonton, AB T6G 2E9, Canada

**Keywords:** dystrophin, Dp71, duchenne muscular dystrophy, isoform

## Abstract

The *DMD* gene is best known for its product dystrophin, a large rod-shaped protein that plays a critical role in muscular membrane strength and integrity. Mutations affecting dystrophin lead to Duchenne muscular dystrophy, a fatal X-linked disease characterized by muscular weakness and breakdown. In addition to the full-length dystrophin product that is most often associated with disease, the *DMD* gene also encodes multiple shorter isoforms of dystrophin with diverse functions. One isoform in particular, Dp71, has been increasingly found to play a wide variety of roles throughout the body. In this narrative review, we consolidate the numerous studies on Dp71 to provide a comprehensive foundation for future work. We outline and summarize the current state of knowledge on the role of Dp71 in the brain, the retina, and skeletal muscles, identifying current knowns and unknowns in the field. We also explore Dp71-based therapies currently being tested in the pre-clinical landscape and identify potential limitations for clinical translation.

## 1. Background

Duchenne muscular dystrophy (DMD) is a debilitating X-linked recessive neuromuscular disorder characterized by progressive degeneration of skeletal muscles [1,2]. Cognitive and neuropsychiatric comorbidities are also prevalent [3]. DMD has a presumed global incidence of around 1 in 3500 to 5000 live male births, with symptoms usually manifesting in early childhood, most often between the ages of 2 and 3 years [4,5]. Loss of independent ambulation typically occurs by 10 to 12 years of age [4,6]. Clinical interventions have improved the natural history of DMD, yet the condition remains life-threatening due to progressive cardiac and respiratory complications, and life expectancy extends into the third or fourth decade [4,6]. The underlying cause of DMD is a mutation in the *DMD* gene located at Xp21.1, which measures approximately 2.4 million base pairs and encodes a protein of 3685 amino acids, making it the largest gene found in the human genome [1,7]. Most pathogenic mutations encompass large deletions or duplications that disrupt the open reading frame, thereby preventing the synthesis of functional dystrophin protein [8,9].

Dystrophin acts as a mechanical link between the intracellular F-actin cytoskeleton and the extracellular matrix through association with the dystrophin-associated glycoprotein complex (DAPC) at the sarcolemma [10,11]. This linkage is fundamental for proper muscle fiber contraction, and its absence leads to sarcolemmal fragility, chronic inflammation, repeated myofiber degeneration, and inability to regenerate [12].

The primary product of the *DMD* gene in skeletal and cardiac muscle is the full-length dystrophin protein, Dp427m [13]. Beyond the 79 exons that collectively encode the full-length dystrophin protein, the locus exhibits considerable genetic complexity, producing multiple tissue-specific isoforms through alternative promoters and differential splicing [2,3]. This genetic flexibility results in several shorter C-terminal variants, including Dp260, Dp140, Dp116, and Dp71 [2,14]. Among these, Dp71 is the second smallest but the most ubiquitously expressed isoform, serving as the predominant dystrophin product in non-muscle tissues such as the brain, kidney, liver, lung, and testis [15]. While traditionally described as absent from skeletal muscle, recent studies have confirmed the presence of both Dp71 transcript and protein in skeletal muscle tissue [16].

Early investigations of dystrophin primarily addressed the full-length protein encoded by a 14 kb mRNA until subsequent identification of distal DMD transcripts revealed shorter isoforms, such as Dp71 [15,17,18]. These studies identified a previously unrecognized transcript initially reported as a 6.5 kb mRNA, encoding a smaller dystrophin-related protein distinct from full-length dystrophin in both structure and tissue distribution, which was later clarified to be 4.8 kb in length [3,15,17,19].

**Figure 1:**
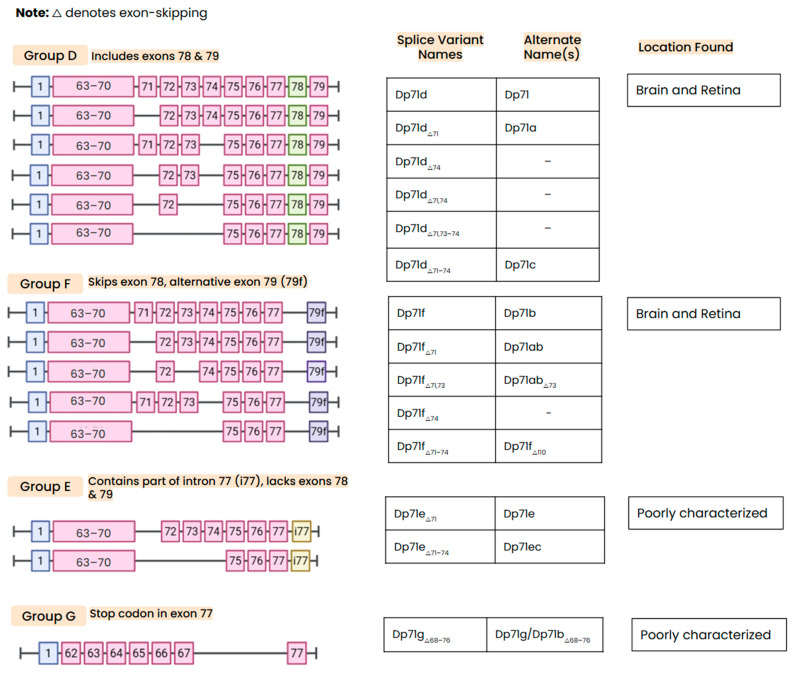
Schematic Representation of Dp71 Splice Variants, Corresponding Nomenclature, and Location. Alternative splicing of the Dp71 transcript generates four major C-terminal variant groups (D–G), distinguished by differences in exon composition. Exon numbering corresponds to the human *DMD* gene transcript. Dp71 and Dp40 share a unique first exon (blue). In Dp71, this exon is spliced to DMD exons 63–79 (pink). Group D contains exons 78 and 79, whereas Group F lacks exon 78, thereby altering the reading frame and producing an alternative exon 79 sequence (79f). Group E lacks exons 78 and 79 and retains part of intron 77 (i77), while Group G contains a premature stop codon in exon 77. Colored regions indicate alternatively spliced sequences: exon 78 (green), alternative exon 79 (purple), and the retained sequence from intron 77 (yellow). To date, Dp71d and Dp71f represent the best characterized Dp71 isoforms in the brain and retina, as evidence supporting expression of the remaining isoforms remains limited. Isoforms are ordered by increasing C-terminal divergence from the canonical Dp71d isoform for clarity.

At the protein level, Dp71 is a 70–75 kDa dystrophin isoform derived from an internal promoter located within intron 62 of the DMD gene [17,18,19,20,21]. Dp71, unlike the complete dystrophin protein, lacks the N-terminal actin-binding domain but retains the cysteine-rich and C-terminal regions, which anchor the protein to the DAPC [3]. Formerly known as apodystrophin-1, Dp71 exhibits functional diversity due to alternative splicing of exons 71–74, 78, and intron 77, which results in different variants with distinct C-terminal sequences and expression patterns [2,22,23]. As a result, the *DMD* gene displays sophisticated organization and regulation by producing structurally unique proteins through independent internal promoters. Fourteen distinct Dp71 splice isoforms have been catalogued to date (Figure 1) [3].

**Figure 2:**
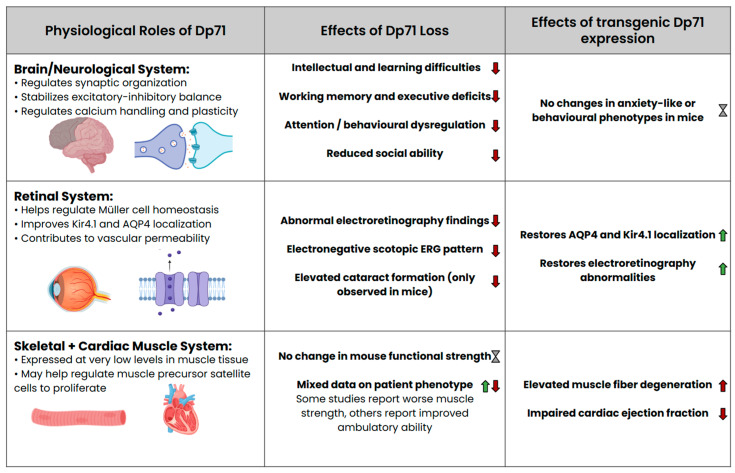
Summary of Downstream Physiological Effects and DMD-associated Phenotypes Linked to Dp71 Disruption. Effects are organized by affected system: brain, retina, and skeletal/cardiac muscle. Downwards red arrows represent detrimental effects, upwards green arrows represent positive effects, and the grey hourglass represents no change. Given the presence of some species-specific findings, effects observed in mice can not be assumed to exist in humans until experimentally confirmed, and vice versa. This figure references data from a variety of studies with different methodologies: Brain/Neurological [26,27,28,29]. Retinal system [30,31,32]. Skeletal and Cardiac Muscle System [33,34,35,36,37].

Dp71 has an important role in the brain and other tissues, and is implicated in the non-muscle manifestations linked to DMD [21,24]. Research using Dp71 reporter mice has confirmed its presence in the adult central nervous system, retina, and kidney epithelium, hinting at roles in neural function, vision, and epithelial–basal lamina interactions [25]. Beyond its structural contributions, Dp71 is actively involved in a range of cellular activities, including neural development, the organization of synapses, the regulation of water balance, and DNA repair mechanisms, all of which are important for maintaining cellular stability and signaling across tissues [3,14]. Dp71 functions through DAPCs in several subcellular compartments, such as the plasma membrane and nucleus, to support cell organization and homeostasis. Disruption of Dp71 expression due to mutations in the distal *DMD* gene is strongly associated with cognitive impairment and other non-muscle features related to DMD, with neurological severity increasing with the loss of shorter isoforms, including Dp71 and Dp140 [3]. This narrative review compiles and summarizes current knowledge regarding Dp71, with a focus on its roles in neurological, retinal, and muscular systems, and evaluates emerging therapeutic approaches. Publications in English involving Dp71 and Dp71-deficient mouse models were manually curated through the U.S National Library of Medicine’s PubMed database. Reference lists from key articles were also screened. No publication date cutoffs were set. A brief visual summary of the systems affected by Dp71 is presented in Figure 2.

## 2. Neurological and Behavioural Roles of Dp71

### 2.1. Preclinical Studies

The primary role of Dp71 pertains to its impact on neurological health and function, and numerous studies have explored the neurological roles of Dp71 in both the clinical and pre-clinical settings. Dp71 is involved in brain maturation, and its expression varies from embryogenesis to adulthood. As the main dystrophin isoform in the adult brain, Dp71 is differentially expressed across tissues, including glial cells, retinal neurons, and kidney epithelia [25,38,39]. In the human embryonic brain, Dp71 is notably active at important stages of growth in the cortex and hippocampus, implying that it is engaged in early brain developmental processes vital for memory and cognition [40]. An expression profile during the human brain development process shows that Dp71 is present in different brain regions and retains expression throughout life, even though levels are lower in the cerebellum [40]. In studies using embryonic rodent brain tissues, it was demonstrated that Dp71 isoforms change over time: Dp71f dominates during embryogenesis, particularly during the expansion of neural stem cells and the formation of glial cells, while Dp71d becomes the primary isoform postnatally [22,41].

Protein interaction investigations in embryonic mouse neural progenitor cells have identified dystroglycan, dystrobrevins, and syntrophins as principal components of macromolecular complexes associated with Dp71 in the developing forebrain [41]. The composition and stability of these complexes are dependent on developmental stage and cell type, indicating that Dp71 fulfills distinct functions during brain maturation. In mouse models, Dp71 is detected in Nestin-positive neural stem and progenitor cells, as well as in radial glia, which provide structural and signaling support during early corticogenesis [41]. Interestingly, while Dp71 expression appears to decline in developing neurons during differentiation, it resurfaces in mature neurons, where it localizes to inhibitory postsynaptic sites. These sites are defined by the presence of gephyrin, a specialized structural protein that anchors inhibitory receptors to the neuron’s membrane [41]. In addition to its action at the synapse, Dp71 also forms DAPCs within the nuclei of hippocampal neurons [42]. Within the nucleus, it resides in nuclear speckles, which regulate gene expression, as well as in the nucleoskeleton, indicating that it is involved in transcription and RNA processing [42]. Collectively, these observations emphasize how Dp71 evolves from important roles within progenitor and glial biology to more specialized tasks in organizing mature neural circuits and forming DAPCs at inhibitory synapses.

#### 2.1.1. Roles in Synaptic Organization and Molecular Compartmentalization

Beyond development, Dp71 fulfills a crucial role as a structural organizer for molecular machinery at synapses, contributing to neuronal communication. In excitatory synapses, Dp71 organizes key postsynaptic proteins, including PSD-95, a major scaffolding protein that anchors glutamate receptors and other signaling molecules to maintain synaptic stability [43]. When Dp71 is absent, as seen in Dp71-null mice, the DAPC is compromised, leading to reduced initiation and maintenance of long-term potentiation (LTP), a process important for synaptic strengthening and memory formation [44]. Further examination of Dp71-null mice at the ultrastructural level reveals abnormalities in presynaptic vesicle positioning, including the displacement of presynaptic vesicles away from the active zone, the enlargement of vesicles in the distal pool, and the widening of synaptic clefts [45]. These structural alterations impair the effectiveness of neurotransmitter release, which corresponds to observed problems in synaptic plasticity, along with the overall stability of neural networks [45].

Recent in vivo compartment-specific studies using an HA-tag knock-in mouse model, in which Dp71 is labeled with a small tag for exact visualization, have allowed for a more comprehensive observation of its location [46]. In dentate granule neurons, Dp71 is found specifically at inhibitory synapses and glial endfeet, but not at the excitatory synapses of CA1 pyramidal neurons [46]. In these mice, Dp71 creates unique glycoprotein complexes at inhibitory postsynapses in dentate granule neurons and at glial endfeet near the blood–brain barrier [46]. These complexes interact with dystrobrevin-β, dystroglycan, and Insyn1 to preserve the structural stability of inhibitory synaptic frameworks. The molecular makeup of these Dp71 complexes is different from that formed by Dp427, suggesting that Dp71 forms distinct complexes in particular synaptic locations.

Furthermore, dystroglycan plays an integral role at the molecular level by anchoring the DAPC to the cell membrane, with Dp71 acting as the specific binding partner [46,47]. Experimental knockdown studies reveal that dystroglycan is strictly required for the proper submembranous localization and stabilization of Dp71, as its loss results in Dp71 dispersing throughout the cytoplasm, and the amount of Dp71 associated with the membrane decreases [41,47].

Lastly, dystroglycan’s interaction with Dp71 facilitates the phosphorylation of Dp71, suggesting that this partnership influences both where Dp71 is located and how it is modified after translation [47]. When this binding is broken, Dp71’s positioning becomes unstable, and its phosphorylation pattern changes, potentially obstructing the proper organization of synapses. Given that dystroglycan complexes mediate membrane–cytoskeleton coupling and extracellular matrix signalling, the absence of Dp71 can give rise to structural instability in both synaptic and glial compartments [41].

#### 2.1.2. Excitation–Inhibition Imbalance in Cortical and Cerebellar Circuits

Basic research using mouse models has highlighted how Dp71 maintains the brain’s electrochemical balance by regulating the interchange between excitatory and inhibitory synaptic transmission. In the medial prefrontal cortex (mPFC), Dp71-null mice manifest enhanced AMPA receptor-mediated excitatory transmission while inhibitory inputs remain intact, leading to an excitation–inhibition (E/I) imbalance [48]. These changes at the synaptic level are linked to issues with spatial working memory and a decline in executive flexibility, which suggests that the coordination of prefrontal networks is compromised [48]. In cerebellar circuits of Dp71-null mice, the lack of Dp71 increases excitatory transmission at the synapses between climbing fibers and Purkinje cells, as shown by larger excitatory postsynaptic currents compared to wild-type controls (about 2.0 nA versus 1.35 nA) [49].

Additionally, the ability of synapses to strengthen or weaken over time, known as activity-dependent synaptic plasticity, is disrupted in the absence of Dp71. In wild-type mice, cerebellar synapses strengthen with spikelet amplitudes, electrical signals that reflect how easily a neuron can be triggered, rising to approximately 126% of baseline 25 to 30 min after climbing fiber (CF) tetanization [49]. Contrarily, Dp71-null mice show no significant change in spikelet amplitude, remaining at baseline levels, which indicates a failure of normal synaptic potentiation [49]. These circuit-level abnormalities translate into behavioural challenges in tasks that rely on the cerebellum. Dp71-null mice showcase slower learning and use less efficient strategies when navigating hidden-platform tests, even though their motor strength and coordination remain normal [46,49].

#### 2.1.3. Calcium Homeostasis

Maintaining proper calcium levels within cells is vital for healthy neurons, and Dp71 appears to play an important role in supporting the protein machinery that controls these levels. A study involving neurons derived from stem cells of a DMD patient with intellectual disabilities revealed that when Dp71 is diminished or mislocalized, it interferes with the proper assembly of the DAPC and its interaction with SERCA2, a calcium pump in the endoplasmic reticulum (ER) responsible for removing surplus calcium from within the cell [50,51]. In cases where Dp71 is absent, neurons exhibit elevated resting calcium levels in their cytoplasm and attempt to compensate by raising SERCA2 expression, denoting a possible link between Dp71 loss and heightened neuronal sensitivity that underpins cognitive impairments [50,51]. Interestingly, even with a rise in SERCA2 expression, the ER still struggles to release calcium effectively, leading to a continual calcium imbalance and poor calcium transmission. Ultimately, these studies demonstrate that a deficiency in Dp71 contributes to disrupted intracellular calcium homeostasis in neurons [50].

Calcium homeostasis is critical for synaptic plasticity, neural excitability, and gene transcription, and disruptions of intracellular calcium have been associated with impaired learning and memory [50,51]. Consequently, the loss or misplacement of Dp71, along with its related DAPC, further compromises membrane protein structures and likely contributes to the observed calcium-mediated signaling issues in mice [43].

#### 2.1.4. Astrocytic and Perivascular Roles of Dp71

**Figure 3:**
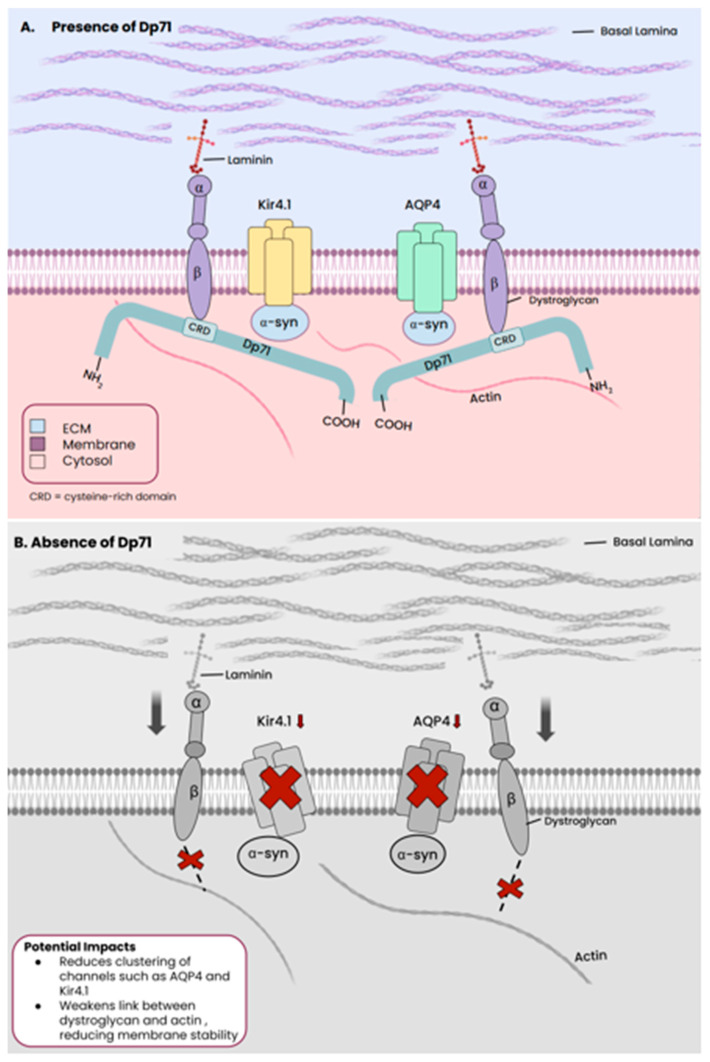
Proposed Model of Dp71 Localization and Protein Interactions. (**A**) At the perivascular astrocytic endfoot membrane, Dp71-associated complexes support the localization of AQP4 and Kir4.1 through interactions with dystroglycan-associated membrane complexes. (**B**) The absence of Dp71 is associated with impaired Kir4.1 and AQP4 clustering. Downward arrows depict reduced localization of both channels at the perivascular membrane, and the red crosses indicate loss of clustering and disruption of membrane organization.

Dp71 is an important constituent of the blood–brain barrier (BBB) and is predominantly localized at the perivascular endfeet of astrocytes [52]. In this location, it has a central role in organizing the DAPC and verifying the correct placement of aquaporin-4 (AQP4) water channels and Kir4.1 potassium channels (Figure 3) [52]. The aforementioned structural arrangement is necessary for maintaining water and potassium homeostasis, supporting the connection between neural activity and blood flow (neurovascular coupling) and controlling neuron excitability. Therefore, if Dp71 is absent, it could upset these regulatory functions, conceivably increasing neural excitability. Studies in mice have indicated that during ischemic injury, Dp71 undergoes breakdown through a process known as ubiquitin-mediated degradation [53]. This degradation is associated with the misplacement of AQP-4 channels, which are necessary for water transport, within the astrocytic endfeet surrounding blood vessels. Consequently, this misplacement contributes to the buildup of interstitial fluid in the brain tissue, worsening swelling referred to as vasogenic edema [53].

#### 2.1.5. Social and Behavioural Abnormalities in Mice

Investigations using mouse models lacking Dp71 have offered insight into the roles of various dystrophin isoforms in motor control, cognitive function, and emotional regulation. For instance, *mdx3cv* mice with a deficiency in both the full-length Dp427 and shorter isoforms, including Dp260, Dp140, Dp116, and Dp71, exhibit a slight delay in operant learning, but overall task acquisition and retention are comparable to wild-type controls [54]. Similarly, their performance in short-term and working memory tests, such as the T-maze, is largely unaffected. In contrast, *mdx* mice, which lack full-length dystrophin but retain Dp71, do not show significant differences from control groups in operant and T-maze assessments [54]. Notably, *mdx3cv* mice present amplified anxiety and more pronounced stress reactions in light–dark tests [54]. While the absence of multiple isoforms in *mdx3cv* mice complicates crediting these behavioural patterns solely to Dp71, a comparison with *mdx* mice reveals that the loss of C-terminal isoforms, including Dp71, can aggravate learning difficulties and anxiety-related behaviors. Furthermore, Dp71-null mouse models display specific impairments in navigation activities reliant on the cerebellum [49]. These mice have slower acquisition and employ less efficient search strategies in hidden-platform spatial tests, despite maintaining normal motor strength, coordination, and general movement [49].

Dp71-null mice also display specific changes in their social and emotional behaviors [55]. While their overall sociability appears normal, these mice show less interest in social novelty, which could suggest difficulties recognizing social cues or reduced social exploratory behaviour. Their socially driven ultrasonic vocalizations are also impacted, with longer calls and a noticeable change in the types of calls they make, leaning more towards peak, sinusoidal, and U-shaped patterns [55]. This points to a disturbance in their social communication. When it comes to emotional responses, Dp71-null mice demonstrate a moderate increase in anxiety-like responses, such as increased thigmotaxis in open-field tests and altered risk assessment in elevated plus-maze and light–dark paradigms [55]. These behavioural changes occur without any evident motor issues, myopathy, or fear-related memory and learning deficits. The observed behavioural profile in Dp71-null mice parallels the descriptions of autistic characteristics and executive function challenges in individuals with distal *DMD* mutations [26].

In brief, preclinical studies in rodent and human embryonic brains demonstrate that Dp71 isoforms are temporally regulated and perform distinct roles in neural progenitors, glial cells, mature inhibitory synapses, and in nuclear gene regulation [22,40]. Dp71 also displays a role in calcium, water, and potassium homeostasis, helping to regulate neural excitability [48,49,50,52]. Thus, the cellular context and timing of Dp71 loss may influence neurological phenotypes. Mouse studies indicate that the severity of neurobehavioural deficits may correspond with the degree of dystrophin isoform loss. *mdx* mice display mild cognitive and behavioural changes, whereas *mdx3cv* and Dp71-null mice show more pronounced phenotypes, including increased anxiety-like behaviour, impaired spatial learning, altered social interactions, and abnormal vocalization patterns [49,54,55]. Interpretation of these outcomes is complicated by the use of multi-isoform mouse models, such as *mdx* and *mdx3cv*, which do not distinguish the effects of Dp71 deficiency compared to the absence of other short isoforms. Additional studies using Dp71-null mouse models can help define the precise consequences of Dp71 loss.

### 2.2. Clinical Studies

#### 2.2.1. Dp71 Disruption and Severe Intellectual Disability

The level of cognitive impairment in DMD is determined by the location of the mutation within the dystrophin gene, with mutations that disrupt the brain-expressed Dp71 isoform acknowledged as a significant predictor of severe intellectual disability [26,27]. Early clinical studies discovered that mutations in the distal C-terminal region are overrepresented in patients with cognitive impairments, underscoring the role of Dp71 and other short isoforms in neurocognitive function [56,57]. The discovery of a three-base pair deletion in Dp71 that results in intellectual impairment without muscular dystrophy substantiates this relationship [58]. Large cohort studies illustrate that individuals with mutations predicted to eliminate Dp71 expression have considerably lower Full-Scale Intelligence Quotient (FSIQ) scores than people with mutations that spare Dp71 [27]. On average, Dp71 deficiency reduces IQ by about two standard deviations, and most affected individuals meet criteria for severe intellectual disability, defined as an IQ below 50 [27,28,59]. Recent multicenter studies provide additional stratification: 64% of patients with mutations downstream of exon 63 that disrupt Dp71 had intellectual disability [26]. This incidence is much greater than the 25% reported in individuals with mutations affecting Dp260, Dp140, or Dp116, and the 15% in those with mutations limited to the long Dp427 isoform. Collectively, these findings highlight the loss of Dp71 as an important contributor to cognitive impairment in DMD.

#### 2.2.2. Dp71-Specific Neurophysiological and Behavioural Impairments

Disruption of Dp71 caused by distal mutations results in numerous cognitive and behavioural impairments. Neuropsychological tests illustrate that boys with distal mutations affecting Dp71 had severe impairments in working memory, verbal reasoning, and reading acquisition, as well as reductions in verbal IQ (VIQ), performance IQ (PIQ), and FSIQ scores [28,59]. Behavioural assessments additionally reveal that Dp71 disruption contributes to poor social and emotional functioning [26]. Elevated scores on the Social Communication Disorder Checklist (SCDC) are observed in DMD patients, with the most pronounced deficits in individuals lacking short brain dystrophin isoforms, including Dp71. These mutations are also associated with the lowest IQ scores and the most impaired working memory within the studied population [26]. Furthermore, the loss of Dp140 and Dp71 is associated with increased rates of neuropsychiatric comorbidities, including attention-deficit/hyperactivity disorder (ADHD), autistic features, and broader executive dysfunction [26,60]. In DMD patients without intellectual disability, subtle impairments in specific cognitive domains, such as implicit learning, may be attributable to dysfunction in cerebro–cerebellar circuits [61]. Although mutations affecting Dp140 and Dp71 are also investigated in this study, the association between implicit learning deficits and these distal mutations was not statistically significant [61]. Overall, these clinical findings show that Dp71 loss is associated not only with intellectual disability, but also with executive and social–behavioural dysfunction.

Collectively, clinical studies have established that Dp71 deficiency is frequently associated with neurocognitive impairments in DMD characterized by lower IQ, difficulties with executive function, and abnormal neurobehavioural assessments [26,27,28,59,60]. However, distal dystrophin gene mutations frequently disrupt several brain-expressed isoforms, such as Dp140, which makes it difficult to isolate the effects of Dp71 alone in human studies. Future longitudinal and genotype-specific studies on Dp71 loss are warranted to determine its independent contributions to neurobehavioural outcomes in DMD. Despite this challenge, assessing patterns of Dp71 loss remains translationally relevant for guiding cognitive screening and intervention for patients with distal gene mutations. A summary of Dp71-related neurological studies is provided in Table 1.

## 3. Dp71 in the Retina

### 3.1. Preclinical Studies

#### 3.1.1. Dp71 Regulates Müller Cell Homeostasis

Although Dp260 is generally regarded as the primary dystrophin isoform in the retina, Dp71 has also been found to be involved in retinal development and function. Dp71 expression in the retina was originally explored following the observation that mice with N-terminal *DMD* mutations displayed more severe electroretinogram (ERG) defects than mice with C-terminal *DMD* mutations, suggesting the involvement of short dystrophin isoforms [62]. Dp71 was found to localize to the inner limiting membrane (ILM) and blood vessels of the retina, whereas Dp260 localizes to the outer plexiform layer, indicating distinct expression patterns and roles. Dp71 in the ILM was further found to co-localize with the dystrophin-associated protein complex (DAPC) in Müller cells, the retina-specific glial cells that are critical for retinal structure and stability [63,64,65]. Dp71 is ubiquitously expressed in all retinal precursors during early embryonic development but becomes layer-specific as the retinal cells differentiate [66].

The primary mechanism of Dp71 in the retina is to regulate the membrane transport of potassium and water, which has direct effects on Müller cell homeostasis and vascular permeability [67]. Dp71 forms a unique DAPC in Müller cells, and this complex is important for the appropriate anchoring and localization of both inwardly rectifying potassium channels (Kir4.1) and the neuronal aquaporin, AQP4 [68]. Thus, Müller cells isolated from Dp71-deficient retinas show impaired potassium conductance [68,69]. Similarly, in mouse models lacking Dp71, the localization and overall expression of Kir4.1 are impaired, while for AQP4, localization is impaired but the overall expression is not affected [68,70,71]. Interestingly, experimental detachment of the retina in mice has also been shown to reduce the expression of both Kir4.1 and Dp71 in isolated Müller cells, suggesting the possibility of bi-directional regulation between Dp71 expression and retinal health [72]. Beyond AQP4 and Kir4.1, Dp71 deficiency has also been associated with elevated vascular permeability of the blood–retina barrier (BRB), as well as impaired retinal vascular development in young mice [72,73]. Mice with a Dp71 deletion have been shown to experience elevated retinal vascular inflammation, vascular lesions, and capillary degeneration, suggesting a possible contribution to retinal vascular disease as well [74].

#### 3.1.2. Dp71 Deficiency Is Associated with Electroretinographic Abnormalities

Electroretinographic studies in Dp71-deficient mice have also established the importance of Dp71 for retinal function. The *mdx3cv* model, which lacks all dystrophin isoforms, was originally demonstrated to show abnormal ERG findings similar to those observed in DMD patients, characterized by reduced b-wave amplitudes [75]. Of note, the lack of all dystrophin isoforms in this model makes it difficult to ascertain whether any change is due to the absence of Dp71 or some other isoform. More recently, studies in Dp71-null mice have explicitly confirmed that this reduction in ERG B-wave amplitude is due to the loss of Dp71, rather than Dp260 [30]. Lastly, Dp71-null mice have been found to show elevated rates of cataract formation, although this is not typically something observed in DMD [31].

### 3.2. Clinical Studies

Clinically, only a single study has explored the contribution of Dp71 to vision defects in patients. Ricotti et al. stratified ocular abnormalities by *DMD* mutation location and found that patients with mutations affecting Dp71 expression showed the most pronounced ERG defects [32]. Specifically, these patients showed a highly electronegative scotopic ERG, even compared to patients lacking Dp260. Given the number of pre-clinical studies that have identified the importance of Dp71 in the retina, future clinical studies should continue to expand upon the findings of Ricotti et al. to better characterize clinical manifestations of Dp71 deficiency on retinal function.

In summary, Dp71 has an important and distinct role in the retina where it regulates Müller cell homeostasis and vascular permeability. This is mediated through the Dp71-dependent regulation of both KiR4.1 and AQP4, contributing to the transport of water and potassium [68]. Consequently, the lack of Dp71 in the retina contributes to retinal dysfunction, detectable as reduced B-wave amplitude on ERG, and may contribute to cataract formation [31,75].

## 4. Dp71 in Cardiac and Skeletal Muscle

### 4.1. Preclinical Studies

Compared to the studies on neuronal and retinal expression, the contributions of Dp71 to skeletal muscle are relatively understudied and occasionally conflict with one another. When it was originally reported, Dp71 was not thought to be expressed in adult skeletal muscle; however, its expression was confirmed in myogenic cell culture from fetal muscle origin [17,18,76]. More recent publications have argued this claim and identified a low level of Dp71 expression in healthy and *mdx* mice, human skeletal muscle, and primary human myocyte culture [16,77]. This discrepancy may arise from an extremely low level of Dp71 expression in mature skeletal muscle, which may only be detectable with the highly sensitive capillary Western blot system used in the more recent studies [16]. The expression of Dp71 in the heart has been confirmed in both humans and mice [78,79].

#### Transgenic Dp71 Expression Exerts a Dominant Negative Effect in Muscle

Several studies have found that transgenic Dp71 expression may exert a detrimental effect in both skeletal and cardiac tissue. It was originally reported that although transgenic Dp71 can effectively colocalize with the DAPC in muscle tissue, muscle pathology remained severely dystrophic, suggesting minimal function in the Dp71-bound DAPC [80]. It was later theorized that skeletal muscle expression of Dp71 may exert a dominant negative effect by competing with full-length dystrophin for DAPC binding sites [33]. Leibovitz et al. found that transgenic overexpression of Dp71 in healthy mice led to muscle damage similar to that of a dystrophic mouse, marked by muscle fiber degeneration and elevated serum creatine kinase [33]. Although there was no change in isometric tension between healthy and transgenic animals, Dp71-overexpressing mice were found to have a higher risk of sarcolemmal rupture [81]. In contrast, it was observed by Lim et al. that healthy mice with transgenic overexpression of human Dp71 demonstrated normal skeletal muscle function, but impaired cardiac muscle function reminiscent of a dilated cardiomyopathy [34]. Transgenic mice displayed reduced ventricular ejection fraction and reduced ventricular wall thickness as early as 3 months of age, and by 12 months, transgenic mice showed elevated systolic volumes and reduced ejection fraction relative to healthy controls [34]. The differing effect of Dp71 overexpression observed by Leibovitz and Lim may arise at least partially from differing methods and magnitudes of overexpression. While Lim used a mouse model overexpressing Dp71 via an integrated human *DMD* transgene, Leibovitz used a plasmid-based system driven by the CMV promoter that would likely show a much higher overall level of Dp71 expression [33,34]. If so, this would suggest that the dominant negative effect of Dp71 on skeletal muscle is dose-dependent and may only arise at very high levels of Dp71 expression.

Functional testing in *mdx52* mice, which only express Dp71, and DMD-null mice, which express no isoforms, revealed no change in grip strength or treadmill performance between the two models, further suggesting a low contribution of Dp71 to these parameters [35]. Interestingly, Dp71 expression—specifically Dp71ab—has been identified in muscle precursor satellite cells, and Dp71ab overexpression was associated with enhanced myoblast proliferation in vitro [82]. These findings suggest that Dp71 may have roles in muscle that do not involve association with the DAPC, and should be explored further.

### 4.2. Clinical Studies

#### Clinical Evidence for the Role of Dp71 in Muscle Is Mixed

Clinical data regarding the importance of Dp71 to skeletal muscle function is mixed. Analysis of patient motor outcomes in a clinical trial setting identified that patients with *DMD* mutations affecting Dp71 expression showed significantly worse performance in the time-to-stand test compared to *DMD* mutations that do not alter Dp71 [36]. However, no other test parameters in this cohort showed a change in severity based on mutation type. A separate study also found that North Star Ambulatory Assessment (NSAA), a measure of motor ability, is significantly reduced in patients with Dp71-affecting *DMD* mutations compared to DMD patients with preserved Dp71 expression [35]. In contrast, genotype–phenotype correlations from a Canadian DMD registry found that patients with Dp71-affecting mutations were significantly less likely to require wheelchair use than other DMD patients [37]. Although the sample size of the Canadian registry was relatively small and the clinical parameters assessed were different, these discrepant results warrant further clinical and pre-clinical studies to better understand the contribution of Dp71 to skeletal muscle function and clinical severity.

Taken together, the multitude of conflicting studies fail to provide a clear picture of the potential roles of Dp71 in muscle. Although recent studies have identified Dp71 in bulk extract from muscle tissue, it is unknown which specific cell populations within might be expressing Dp71. Given that satellite cells have been shown to express Dp71 themselves, further studies should clarify whether the Dp71 signal observed in muscle tissue arises purely from satellite cells or whether it is also expressed in the mature myotubes [82]. The lack of a difference in grip strength or treadmill running between mice with or without Dp71 suggests that the primary role of Dp71 in muscle is unlikely to involve interactions with the DAPC in the contractile muscle unit [35]. This is further supported by the impaired function and sarcolemmal rupture that arises when Dp71 is overexpressed at a high rate in muscles [33,81]. However, the impaired outcomes in patients lacking Dp71 suggest that Dp71 still has an important role in skeletal muscle, even if it is not directly related to muscle contraction [35,36]. Future work should continue to explore this phenomenon, as well as aim to clarify the contrasting results found by many groups at both the basic and clinical levels.

## 5. Emerging Dp71-Based Therapies

While no Dp71-specific therapies have yet been translated into clinical trials, several studies have explored the preclinical therapeutic utility of Dp71 overexpression. Given the retinal and cognitive deficits observed due to the lack of Dp71 expression, it was theorized that Adeno-associated virus (AAV)-mediated delivery of a Dp71 transgene may serve as an effective therapy to treat these symptoms. Uptake of AAV into Müller cells is typically very low, so early studies focused on the development and optimization of AAV serovariants that displayed elevated Müller cell uptake [83]. Using directed evolution, a variant called AAV ShH10 was identified that showed effective and selective uptake into Müller cells following intravitreal injection [83]. Interestingly, AAV-ShH10 showed higher transduction efficiency in the Müller cells of Dp71-deficient mice compared to healthy mice, possibly due to membrane damage [84].

Dp71-null mice treated via intraretinal injection with Dp71 transgene packaged in AAV-ShH10 showed significant expression of Dp71 transcript and protein that was specific to Müller cells, with higher expression than both untreated Dp71-null mice and WT mice [85]. This overexpression was associated with significantly elevated AQP4 and Kir4.1 expression in treated mice, and the restored localization of these proteins to Müller cell endfeet. Permeability of the BRB was also restored to WT levels in treated mice [85]. A follow-up study later identified that this treatment was associated with improved ERG performance [86]. Dp71-null mice generally show impairment on ERG characterized by reduced b-waves and smaller response amplitudes. Compared to sham-treated controls, Dp71-null mice treated with intraretinal ShH10-Dp71 showed a fully recovered ERG response consistent with healthy mice [86]. These findings show that Dp71-based therapies, specifically viral transgene delivery, may be an effective tool for addressing vision defects in patients with Dp71 deficiency. Most recently, the Dp71 transgene was packaged in an AAV9 vector and delivered to neonatal Dp71-null mice through intracardiac injection, which led to significantly elevated Dp71 expression in the hippocampus, cerebral cortex, and cerebellum [29]. However, treatment failed to show any benefit in behavioural or anxiety-like symptoms measured through light–dark choice testing, elevated maze testing, or open field testing.

Collectively, these studies show that Dp71 can be effectively and selectively overexpressed in the retina, leading to restored KiR4.1 expression, restored AQP4 expression, and recovery in ERG deficits, but that expression in the brain fails to address the behavioural phenotype in Dp71-null mice [29,85]. This suggests that Dp71 overexpression may have therapeutic value for the retinal defects of DMD, but not for the cognitive defects. However, several barriers exist that must be overcome prior to clinical translation of a Dp71-based therapy. Given the potential dominant negative effects associated with muscular and cardiac expression of Dp71, the risks of systemic or off-target Dp71 expression must be carefully assessed [33,34]. A prior study identified that the BRB in Dp71-null mice remains impermeable to ShH10-GFP despite the known BRB damage in this model, offering some evidence that intravitreal injection will not lead to systemic transgene expression; however, this must be validated in humans prior to any translation [83]. In addition to the Dp71 transgene, the safety profile of the selected AAV vector must also be explored. Although AAV-based clinical trials have shown high success in numerous recent trials, there have also been a growing number of concerns about the safety of these therapies [87]. Although rare, fatal AAV-related toxicity has been observed in five patients across four different trials in recent years [87]. Furthermore, treatment with an AAV-based therapy can activate the immunological production of anti-AAV antibodies, precluding the use of additional AAV-based therapies [88]. This may conflict directly with AAV-delivered microdystrophin, a different transgenic therapy for DMD that has seen high amounts of research and clinical support in recent years [11,89,90]. Continued studies in this field should consider and address these concerns.

## 6. Conclusions

Dp71 has been shown to play a steadily increasing number of diverse roles throughout the body. It is critical for neuronal development and synaptic function, and the absence of Dp71 is associated with both cognitive and behavioural deficits. It also plays a key role in retinal health and function, and the loss of Dp71 is a contributing factor to impaired vision and retinal vascular health. Finally, Dp71 is implicated in a growing number of interactions with skeletal muscle; however, the data are mixed on whether the presence of Dp71 in muscle is a net benefit or a detriment to muscle strength and function. Future studies should aim to clarify this discrepancy, particularly by exploring the expression and contributions of Dp71 to muscle satellite cells versus the muscle contractile unit itself. Lastly, Dp71-based therapies have been explored and validated at the preclinical stage, but consideration must be given to the potential safety concerns associated with systemic Dp71 expression and competition with AAV-delivered microdystrophin.

## Figures and Tables

**Table 1:** Summary of Neurological and Behavioural Studies on Dp71.

Level	System	Dp71 Alteration	Main Findings	Translational Relevance	Reference(s)
Human	DMD clinical cohorts	Distal mutations affecting the Dp71 region	Lower IQ, impaired working memory, language/executive dysfunction, increased ADHD/autistic traits	Distal DMD mutations predicted to affect Dp71 are associated with increased risk of cognitive impairment	[26,27,59,60]
Human	Patients with isolated Dp71 disruption	Dp71 mutation with preserved full-length dystrophin	Intellectual disability (ID) without muscular dystrophy	Suggests Dp71 dysfunction can contribute to cognitive impairment independent of full-length dystrophin deficiency	[58]
Human	Non-ID DMD cohorts	Distal mutations affecting the Dp71 region (including the Dp140 region)	Subtle cognitive differences (e.g., implicit learning), distal mutation site not statistically significant	Supports possible mild effects on certain cognitive functions, but overall evidence is limited	[61]
Mouse	Dp71-null mice	Complete loss of Dp71	Synaptic disorganization, reduced LTP, E/I imbalance, impaired working memory, increased anxiety-like and altered social behaviours	Findings appear consistent with executive dysfunction and anxiety-related features observed in DMD	[48,49,55]
Mouse	*mdx* and *mdx3cv* mice	Loss of Dp427 alone (*mdx*) or Dp427 + short isoforms including Dp71 (*mdx3cv*)	*mdx* mice show relatively preserved cognition, whereas *mdx3cv* mice show greater learning and stress-related abnormalities	Suggests that the cumulative loss of short dystrophin isoforms, including Dp71, aggravates neurobehavioural outcomes, although Dp71-specific effects cannot be isolated	[54]
Mouse	Mouse model of middle cerebral artery occlusion	Dp71 degradation at astrocytic endfeet	Mislocalized AQP4/Kir4.1, disrupted water/ion homeostasis, worsened edema after injury	Associates the loss of Dp71 with disrupted blood–brain barrier function and astrocyte endfeet organization	[53]
Human + Mouse developmental studies	Brain	Developmental regulation of Dp71 expression	Dp71 is expressed in the human embryonic cortex and hippocampus, in mouse neural stem cells and radial glia, with isoform expression changing across neurodevelopment	Supports a role for Dp71 in early brain development and neural circuit formation	[22,40,41]
Cellular	Neurons derived from a DMD patient with ID (iPSC-DMD)	Reduced/mislocalized Dp71	Elevated intracellular Ca^2+^, impaired ER calcium handling, disrupted DAPC–SERCA2 interaction	Dp71 loss may participate in disrupted calcium homeostasis and neuronal excitability	[50]

## Data Availability

No new data were generated as part of this review article.
